# Crowding types in high-density tourist cities: Based on a physical and-perceived density clustering analysis

**DOI:** 10.1371/journal.pone.0360676

**Published:** 2026-11-12

**Authors:** Shuting Wang

**Affiliations:** College of Architecture and Art, Hebei University of Engineering, Handan, Hebei, China; IULM: Libera Universita di Lingue e Comunicazione, ITALY

## Abstract

Driven by global urbanization and the rise of mass tourism, crowding in high-density tourist cities has become a significant concern in the academic community. However, existing studies often lack a systematic categorization of crowding types, resulting in insufficiently targeted governance strategies. In this study, a two‑dimensional framework of physical and perceived crowding is employed to construct a comprehensive indicator system comprising eight physical crowding indicators and six perceived crowding indicators. Furthermore, twenty‑eight high‑density urban areas worldwide are selected, and cluster analysis is applied to identify crowding types and examine the key influencing factors of each type. The results present four crowding types: closely spaced narrow alley, high building quantity, high‑rise low‑greenery, and high vehicle interference. Additionally, correlation analysis reveals the interaction mechanisms that exist among factors. Building coverage is positively correlated with road network density. Moreover, building spacing is positively correlated with both the building height and the building height‑to‑width ratio. Façade color is positively correlated with the road network density. Finally, the number of vehicles within an area is positively correlated with both the road width and site area. Conversely, building coverage is negatively correlated with the building spacing, depth‑to‑height ratio (D/H), and green space ratio. In addition, building spacing is negatively correlated with the number of buildings within an area, and façade color is negatively correlated with the green space ratio. This research enriches the evaluation indices considered in both the physical and perceived dimensions. The four identified types of crowded spaces derived from empirical analysis can provide a scientific basis for developing future differentiated planning strategies.

## 1 Introduction

With the acceleration of global urbanization, the crowding problem arising from excessive population concentration has become a focal concern in academic discourse [1]. In November 12, 2020, approximately 55% of the global population resided in urban areas [2]. The rapid expansion of tourism has further exacerbated urban crowding through the massive influx of transient and floating populations. According to the United Nations World Tourism Organization (UNWTO), international tourist arrivals exceeded 285 million in the first quarter of 2024, indicating an increase of approximately 20% compared with the number in the same period of the previous year [3]. Notably, 70% of the tourist population is concentrated in the top 50 tourist cities. In destinations such as Venice and Kyoto, the instantaneous population density during peak seasons can reach 5 to 10 times that of the permanent resident population [4]. Furthermore, the proliferation of tourism gentrification has caused tourism crowding to impact not only tourist groups but also local residents [5]. First, behavioral friction intensifies spatial use conflicts. The spatiotemporal overlap between tourist behaviors, such as prolonged photo‑taking and sightseeing at bus stops, and resident daily activities, such as commuting and socializing, reduces the efficiency of spatial utilization and increases the probability of social friction. Second, everyday spaces are encroached upon by souvenir shops, social media-famous dining venues, and other tourism-oriented businesses, while tourists displace and crowd the spaces that residents rely on for daily life (e.g., wet markets and community squares). This sense of spatial deprivation directly amplifies the subjective crowding experience. Third, soaring rents and rising living costs driven by gentrification have led to the continuous influx of real estate developers and middle-class residents, forcing the original low- and middle-income residents to relocate [6]. The superposition of these three forces not only exponentially increases crowding perception within a physical space but also further intensifies tensions in a social space.

Owing to accelerating global urbanization and increasing mass tourism demand, the crowding problem in high-density tourist cities has evolved from a localized phenomenon to a widely recognized concern. The complexity of urban crowding lies in its dual nature as both a spatial and sociopsychological phenomenon [7]. This overload results in physical crowding and creates negative emotions among individuals [8]. Excessively high physical density may lead to hard conflicts such as infrastructure overload, public space encroachment, and traffic obstruction, whereas an imbalance in perceived density triggers soft conflicts such as heightened tourist oppressiveness and resident resentment [9]. In high-density tourist cities, the superposition of these two effects is even more pronounced. Therefore, alleviating crowding and enhancing the comfort of both tourists and residents constitute critical challenges and prominent research issues. Existing research on crowding is organized primarily around two core components: psychological crowding and physical crowding. Studies on psychological crowding are focused mainly on the factors influencing crowding, the relationship between crowding and density, and the connection of crowding to loyalty and satisfaction [10–16]. Physical crowding research is focused largely on the spatial and environmental elements associated with crowding perception, tourism carrying capacity, and tourism capacity [17,18]. Although these studies have yielded substantial findings in identifying the causes and analyzing the effects of crowding, a systematic classification of crowding types remains absent. The causes of crowding vary across cities. Without differentiating these distinct causes, precise improvement measures cannot be implemented.

Accordingly, in this study, a two-dimensional framework of physical density and perceived density is adopted. This framework is used to construct an evaluation system comprising eight physical crowding indicators and six perceived crowding indicators. Twenty-eight high-density tourist city areas worldwide are selected, and cluster analysis is applied to identify crowding types and elucidate the key influencing factors of each type. Considering this objective, three research questions are addressed herein: (1) What typical crowding types can be identified in high-density tourist city areas on the basis of the two-dimensional indicators of physical density and perceived density? (2) How do the core spatial characteristic factors of each crowding type uniquely influence crowding perception? (3) What correlations exist between key physical density indicators and perceived density indicators? By answering these questions, the problem of poorly targeted governance strategies arising from a lack of systematic classification of crowding types can be addressed, thereby providing a scientific basis for future research and management practices.

## 2 Literature review

### 2.1 Physical and perceived crowding density framework

This study employs cluster analysis to categorize crowding types in high-density tourist cities to establish a theoretical framework for research on crowding perception. According to a literature review, many scholars have sought to explain the mechanisms underlying crowding perception. The most authoritative explanatory theories include the following three. (1) Stimulus overload theory [18], which was proposed by Simmel and Wirth, is grounded in urban sociology and posits that crowding perception arises when the environmental input to which individuals are exposed exceeds their tolerance [19–21]. Specifically, crowding perception arises when the environment provides excessive stimulation and pressure, leading to negative psychological and perceptual responses [22]. (2) Ecological perspective theory posits that crowding is fundamentally a resource management issue [23]. When the number of individuals exceeds the environmental capacity, the resulting constraint on remaining spatial resources leads to the perception of crowding [24]. Additionally, certain environmental elements weaken the overall carrying capacity and thus contribute to the perception of crowding. These two theories clearly correspond to the two broad dimensions of physical and perceived crowding, which currently constitute the most widely utilized evaluation framework in crowding perception research. Typically, physical crowding is closely associated with capacity issues, whereas perceived crowding is intrinsically linked to individuals’ emotions [25]. (3) Social interference theory was derived from Brehm’s psychological reactance theory and Sommer’s conceptualization of personal space [26,27]. According to this theory, the presence of others restricts the range of behavioral choices, interferes with the goals of an individual, and diminishes their behavioral freedom, thereby contributing to the experience of crowding. The feelings of crowding arising from social interference are attributed to the influence of others and are therefore beyond the scope of this study. On the basis of these considerations, the evaluation framework of this study is focused on both physical and perceived crowding. Factors related to environmental capacity are classified as physical crowding, whereas factors associated with stimulation and emotions are classified as perceived crowding.

### 2.2 Factors influencing crowding perception

#### 2.2.1 Spatial factors affecting perceived crowding.

The height, height-to-width ratio, and depth-to-height ratio (D/H) of a building are key characteristics of the three-dimensional spatial form. These factors directly influence visual perception through sensations of spatial closure and oppression, which can trigger psychological pressure and feelings of crowding. In contrast to the horizontal expanse of low-rise buildings, high-rise structures create a sense of visual oppression in the vertical dimension. The stacking of vertical volumes compels individuals to confront more unnatural geometric forms. John Zacharias and Arthur Stamps investigated the relationship between building spacing and height. Their study indicated that an increase in building height increased crowding perception; however, greater building spacing could mitigate the oppressive feeling associated with increased height to some extent [28]. The impact of D/H on crowding perception has been explored in various studies. For instance, Jaecheol Kim utilized D/H as a variable to examine its effect on the shopping experiences of tourists, whereas Tahir Albayrak et al. analyzed the mechanisms through which spatial elements, including D/H, influence the crowding perceptions of tourists in a closed plaza [29,30]. When D/H > 2, the space is perceived as open and sparse; when 1 < D/H < 2, the spatial scale aligns with ergonomic comfort standards; and when D/H < 1, a pronounced sense of spatial enclosure is experienced [31]. Esmaeil Zarghami et al. evaluated the mechanisms through which changes in the form of high-rise buildings affect a citizen’s sense of oppression. The height-to-width ratio of a building determines whether it is short and wide or tall and slender. As the height-to-width ratio increases, the sense of oppression experienced by individuals increases. When the width is held constant, increasing the height-to-width ratio from 2 to 3 leads to a more pronounced increase in feelings of oppression than increasing it from 3 to 4. The scholars indicated that changes in building height have a more significant effect on the feeling of oppression than changes in width [32]. Additionally, the researchers emphasize that the presence of sufficient greenery can mitigate the oppressive effects of building forms.

The configuration factor of a building refers to the complexity of its appearance. As complexity increases, the visual information load, which is characterized by dynamic contours, uniquely shaped structures, and intricate decorations, intensifies, thereby increasing sensory processing pressure [33]. Color is among the most significant sources of visual stimulation, and bright, saturated colors can lead to visual fatigue and increased psychological pressure. Masaaki Takei and Masaki Ohara conducted two experiments. Experiment 1 verified the relationships among the configuration factor of a building, the horizontal distance between the observer and the building, and the feeling of oppression. The scholars concluded that V = 6.63c^1.21^D^0.42^, where V is the sense of oppression, c is the configuration factor of a building, and D is the horizontal distance. These findings demonstrated that the configuration factor of a building and the sense of oppression of an individual are positively correlated. Experiment 2 explored the effect of façade color on the sense of oppression and concluded that strong colors may cause a building to appear incongruous with its surroundings, thereby increasing the sense of oppression [34]. In contrast, colors with high brightness and low saturation can alleviate one’s sense of oppression. The correlation between spatial perception and color has been confirmed by Arthue E. Stamps as well [35].

Within the stimulus overload framework, greenness acts as a palliative moderator, indirectly alleviating psychological anxiety caused by environmental overload through softening visual boundaries, such as by buffering the harsh lines of buildings through trees, and reducing the intensity of sensory stimuli, such as by implementing green vegetation for its calming effect. Morteza Asgarzadeh et al. investigated the relationships among the views of buildings, trees, the sky, and ground surfaces and their effects on feelings of oppression and spatial perception in a Tokyo urban environment. The results indicated that tall buildings contribute to a heightened sense of oppression, whereas trees play a crucial role in mitigating this feeling [36]. Elke van der Meer et al. examined two spatial factors, namely, vehicles and greenery, to assess the effect of qualitative environmental factors on the sense of crowding [37]. Consequently, the configuration factor, height, depth-to-height ratio (D/H), façade color, green space ratio and height-to-width ratio of a building are defined as elements related to perceived crowding.

#### 2.2.2 Spatial factors affecting physical crowding.

When the numbers of buildings and vehicles in an area exceed the optimal spatial carrying capacity, tourists inevitably perceive crowding. The same principle applies to high building coverage ratios, as these factors compress the usable area and pedestrian flow space of an environment. Huang Yiru and Zhu Peidong reported that perceived density can be expressed by the following equation: perceived density = −0.724 + 0.03 × number of buildings + 0.915 × surface density – 1.249 × (gap sunlight intensity / number of buildings). This equation demonstrates a positive relationship between building count and perceived density [38]. Morteza Asgarzadeh et al. reported that excessive building coverage increases negative feelings and crowding perception [36]. Beatrix Emo et al. analyzed images using six parameters, namely, visibility, number of buildings, street width, sky area, green space area, and number of vehicles, to explore the influences of these key factors on perceived urban density [39]. The results revealed that participants identified the number of vehicles, number of buildings, visibility, and green space as the primary criteria for assessing perceived crowding. The site area directly determines the carrying capacity of a location; smaller sites are more likely to reach or exceed their physical carrying limit (i.e., the threshold of people per unit area), thereby triggering perceived crowding. Morteza Asgarzadeh et al. also reported that a larger site area is effective at alleviating feelings of oppression [36].

Building spacing refers to the gaps between buildings, such as courtyards and alleys, which serve as potential buffer and circulation spaces. Insufficient spacing restricts the area available for movement and gathering between buildings, thereby increasing pressure on spatial resources. John Zacharias and Arthur Stamps conducted experiments demonstrating that variations in building spacing significantly influence perceived crowding [28]. The participants reported a decrease in perceived building density as the degree of building spacing increased. This finding indicates that increasing building spacing may lead to a reduction in perceived building density, potentially alleviating oppression and crowding.

The road width refers to the physical dimension of the access space. A narrow road significantly limits the throughput of people or vehicles within a given time frame, often creating bottlenecks that lead to congested and slow-moving crowds. This situation directly reduces the available transportation capacity and fosters a sense of competition for these resources (e.g., pedestrians must move aside, and vehicles must wait). Elke van der Meer et al. employed the residential satisfaction model as a theoretical framework to systematically analyze the mechanisms through which people perceive and assess various environmental attributes [37]. Among these attributes, the number of vehicles and road width serve as critical factors in the evaluation process, influencing the environmental perception of residents.

Road network density (D_l_) is defined as the total road length (L)/area size (S) [40]. A low road network density can lead to crowding on a limited number of roads, reducing the efficiency and selectivity of spatial resource distribution and increasing the likelihood of localized congestion. Junyan Yang and Beixiang Shi examined the factors influencing building density in the central areas of high-density cities in Asia. The scholars reported that neighborhoods with high road network density enjoy significant advantages, including improved traffic flow, enhanced street vitality, and reduced crowding perception [41]. The selectivity of tourist paths refers to the quantity and degree of optionality regarding sightseeing and circulation routes within designated areas (e.g., tourist attractions or neighborhood blocks). Low optionality indicates that tourists are highly guided or restricted to a few fixed paths (undersupply), resulting in a high concentration of people on these paths and a significant increase in the pressure per unit path length or area. This situation is highly likely to exceed the design capacity of the paths and create crowding hotspots. Juan Guo considered the Wutai Mountain Scenic Area as an example and reported that the fixed and singular nature of tourist routes leads to crowding at various tourist nodes and that the passenger flow from other connected scenic areas is affected [42]. Therefore, the road width, road network density, building coverage ratio, building spacing, building count within the area, site area, tourist path selectivity, and vehicle count within the area are taken as physical crowding-related factors.

### 2.3 Summary of the literature

In summary, existing research has yielded substantial findings in the theoretical elaboration of crowding perception and the identification of influencing factors, providing a solid theoretical foundation for constructing a two-dimensional evaluation framework of physical and perceived density. However, the following limitations remain. First, there is a lack of systematic classification of crowding types. The vast majority of studies concentrate on single causes or isolated effects of crowding, and no research to date has classified crowding types on the basis of multiple crowding factors. Second, comparative research on the core characteristics of different crowding types is insufficient. Existing findings are focused mostly on the relationship between specific spatial elements and crowding perception. However, few researchers have conducted cross-case, multivariable comparisons within a unified framework to reveal which specific combinations of factors drive the crowding types observed in different urban spaces. Third, existing research has not clarified whether physical density indicators and perceived density indicators mutually influence each other. To date, very few studies have explored the associations among various crowding factors—for instance, which indicators reinforce each other, which offset each other, and the direction and strength of these relationships. Therefore, on the basis of the dual dimensions of physical and perceived density, high-density tourist urban areas are classified into distinct types, and the core influencing factors of each type and the mechanisms through which these indicators interact are examined.

## 3 Methodology

### 3.1 Research workflow

The experimental procedure of hierarchical clustering comprises four key steps: study area selection and data collection, data preprocessing and cluster analysis, intercluster distance definition and merging criterion, and determination of the optimal number of clusters and type naming. The details of each step are as follows:

(1) Study area selection and data collection

Twenty-eight high-density tourist city areas were selected, encompassing building-dense and pedestrian-intensive locations such as commercial centers, historic districts, and transportation hubs, while ensuring the representation of diverse functional types, including commerce, culture, and leisure sightseeing. Physical crowding indicators, namely, building count, road width, and road network density, were extracted using QGIS (https://www.qgis.org). The building spacing, depth-to-height (D/H) ratio, and green space ratio were measured using Google Earth satellite imagery (https://www.google.com/maps). For perceived crowding indicators, building façade colors and spatial enclosures were assessed on the basis of Baidu and Google Street View images (https://www.google.com/streetview). Simultaneously, a 5-point Likert scale questionnaire was distributed through the Wenjuanxing platform (www.wjx.cn) to collect the subjective ratings of oppressiveness and perceived crowding of tourists.

(2) Data preprocessing and cluster analysis

All continuous variables were standardized to z scores (mean = 0, standard deviation = 1) to eliminate dimensional effects. Hierarchical agglomerative clustering was performed in a bottom-up fashion. First, each sample was treated as an individual cluster. Then, the two closest clusters were merged iteratively until all the samples were combined into a single cluster. Euclidean distance was adopted as the distance metric [43], and a distance matrix was calculated accordingly. In the first step, the two closest individuals were merged. The formula could be expressed as follows:


$$\begin{array}{c} \begin{array}{c} d(A,B)=\sqrt{\sum_{i=\text{1}}^{p}(x_{Ai}-x_{Bi}{)}^{\text{2}}} \end{array} \end{array}$$ (1)

where $x_{Ai}$ denotes the specific value of sample A on the i-th variable, $x_{Bi}$ denotes the specific value of sample B on the i-th variable, and p is the total number of variables or features.

(3) Intercluster distance definition and merging criterion: Ward’s minimum variance method

In this study, Ward’s minimum variance method was adopted as the linkage criterion for cluster merging [44]. The specific procedure could be expressed as follows. First, the internal compactness of any given cluster (e.g., Cluster C) was measured by the within-cluster error sum of squares (ESS), which was calculated as follows:


$$\begin{array}{c} ESS(C)=\sum_{i\in C}{\left\|x_{i}-{\bar{x}}_{C}\right\|}^{2} \end{array}$$ (2)

where $x_{i}$ is a sample point in the cluster and ${\bar{x}}_{C}$ is the centroid of that cluster. A smaller ESS indicated greater homogeneity among the samples within the cluster.

Next, for Clusters A and B, the Ward distance Δ was computed as follows:


$$\begin{array}{c} \begin{array}{c} \Delta (A,B)=ESS(A\cup B)-[ESS(A)+ESS(B)] \end{array} \end{array}$$ (3)

where ESS(A) and ESS(B) are the within-cluster error sums of the squares of Cluster A and Cluster B, respectively, before merging, and ESS(A∪B) is the within-cluster error sum of the squares of the temporary cluster formed by combining all the samples of Cluster A and Cluster B. A smaller Δ value suggests that the original structures of the two clusters are more similar, and the merger is thus more justified.

(4) Determination of the optimal number of clusters and type name

Once the clustering procedure is complete, a dendrogram can be constructed on the basis of the sequential merging order and the corresponding Ward increments. However, the dendrogram alone does not indicate the number of clusters to retain. To determine the optimal number of clusters, the elbow method and the Calinski–Harabasz index (CHI) were used in combination. The elbow method was used to identify the optimal number of clusters by locating the inflection point where the rate of decrease in WCSS markedly slowed. The CHI was used to evaluate clustering quality by computing the ratio of between-cluster to within-cluster dispersion; specifically, larger values indicated better clustering performance [45–47]. By integrating both criteria and considering classification interpretability, the final number of clusters was determined, and the dendrogram was cut at the corresponding height, yielding the crowding type for each area. Finally, overarching type names were assigned on the basis of the characteristic distributions of each cluster across the physical and perceived crowding indicators, with reference to the literature.

### 3.2 Evaluation metrics for crowding cites

The data acquisition and calculation methods for each factor, as organized from the preceding review, are summarized in Table 1. The relevant indicator data were obtained through multiple approaches, including Google Earth satellite image measurement, QGIS software extraction, and Baidu/Google Street View image analysis. All the data were original, and the collection and analysis methods complied with the terms and conditions of the respective data sources. To ensure the reliability of spatial data for physical morphology indicators, including building spacing, road width, the building coverage ratio, and site area, 36 candidate sites were initially identified during the case selection stage. By evaluating the clarity of satellite images for each site, areas with insufficient resolution or blurred feature edges that precluded accurate interpretation were excluded, resulting in 28 valid cases with high-resolution imagery. For all retained cases, satellite images acquired between 2023 and 2024 were uniformly selected, with a preference for spring or autumn scenes featuring low cloud cover and minimal extensive shadowing, to ensure clarity in feature boundary identification and measurement. For dynamic indicators, such as the number of vehicles within an area, the temporal window was restricted to 10:30–11:30 a.m. or 2:00–3:00 p.m. local time on working days to minimize data variability caused by differences in sampling moments. These intervals corresponded to periods outside peak commuting hours and late-night parking phases, thus providing a relatively stable estimate of the regular daytime motor vehicle load. Moreover, to mitigate the random bias inherent in any single satellite image, all the indicators were cross-validated against co-located and co-temporal historical imagery from Baidu Street View and Google Street View, and anomalous extreme values were simultaneously removed, thereby enhancing cross-regional comparability and data reliability.

**Table 1:** Factor data acquisition.

Dimension	Factor name	Calculation method	Data acquisition method
Physical crowding-related factors	Road width	Average road width in the area	Google Earth measurement collection
	Road network density	*D*_*l*_ *= L/S* (D_l_) is the road network density (L) is the total length of roads (S) is the area of the region	Google Earth measurement collection
	Building coverage ratio	$BCR=\frac{\sum A_{footprint}}{A_{site}}\times \text{100}\%$ ${(A}_{footprint})$ is the total area of the building base ${(A}_{\text{site}})$ is the total area of land	QGIS recognizes the building outline of satellite image
	Building spacing	Average building spacing in the area	Google Earth measurement collection
	Building count within the area	Total number of buildings in the area	QGIS recognizes building outlines in satellite images
	Site area (km^2^)	Size of the selected area	Google Earth measurement collection
	Selectivity of tourist paths	Multiple routes are assigned a score of 1, while a single route is assigned a score of 0.	Google Earth Recognition
	Vehicle count within the area	Total number of vehicles in the area	Google Earth measurement collection
Perceived crowding-related factors	Configuration factor of the building	The configuration factor of the building is defined as the ratio of the solid area of its outer wall to its projected area	Google Earth measurement collection
	Building height	Average building height in the area	Google Earth measurement collection
	Depth-to-height ratio (D/H)	(D) is the horizontal distance between buildings on both sides of the street (H) is the height of buildings on both sides of the street expressed as the average height of buildings on both sides or the representative height of the street-facing buildings.	Google Earth measurement collection
	Façade color	Likert scale to collect the extent to which the color of the area is oppressive to tourists	Questionnaire survey
	Green space ratio	$GGR=\frac{\sum A_{green}}{A_{site}}\times \text{100}\%$ ${(A}_{green})\;$is the total green space ${(A}_{site})$ is the total land area of the area	QGIS extracted green vegetation coverage ratio
	Building height-to-width ratio	The ratio of the building height to its width	Google Earth measurement collection

Questionnaire scales are widely used in environmental perception and color psychology research to determine the visual experiences and emotional feedback of respondents when considering building colors [48]. The questionnaire employed a 5-point Likert scale consisting of three items: (1) To what extent do the building façade colors in this area cause visual fatigue? (1 = no fatigue at all, 5 = extreme fatigue); (2) To what extent do the building colors in this area generate a sense of oppressiveness? (1 = no oppressiveness at all, 5 = extreme oppressiveness); (3) How would you rate the harmony of the building colors in this area? (1 = very harmonious, 5 = very chaotic). The mean score of the three items served as a composite indicator of the visual effect of building façade color in the area. The questionnaire was distributed anonymously via both online and offline channels.

### 3.3 Case study

During case selection, multiple factors were considered: pedestrian traffic density, type diversity, regional representativeness, and data availability. First, priority was given to urban core areas or famous tourist destinations with high building density and heavy pedestrian flow. For example, the Colosseum in Rome, a world-class cultural heritage site, attracts more than 20,000 visitors daily on average. Asakusa Temple in Tokyo received 2.9 million visitors in January 2020. Times Square in New York, Causeway Bay in Hong Kong, Umeda in Osaka, and other commercial hubs exhibit significant pedestrian flow peaks, a typical feature of high-density urban areas. To avoid sample homogeneity, cases were selected to cover a wide range of areas, including historical and cultural landmarks, modern commercial centers, distinctive neighborhoods, bazaars, and natural recreational sites, thereby comprehensively capturing differences in crowding perceptions across environments. Special consideration was given to the impact of tourism gentrification on urban spatial structure, encompassing two types of areas. The first type comprises areas with gentrification potential, which are typically located in city centers and characterized by a concentration of low-income residents and underinvestment. Examples include Edinburgh’s Grass Market, which attracted low-income residents after the decline of its historic market, and the area around the Rialto Bridge in Venice, where the community is gradually hollowing because of intensifying tourism and commercialization, leading to the outward migration of local residents. The second type comprises areas already undergoing gentrification, characterized by an influx of affluent groups and increased capital investment. Examples include Covent Garden in London, which has transformed from a former warehouse district into a high-end commercial and cultural landmark; Wall Street in New York, which remains upmarket because of the concentration of financial elites; and Shinsaibashi in Osaka, which has evolved from a traditional shopping street into a trendy consumer landmark through capital injection [49]. Similar capital-driven spatial restructuring and urban fragmentation have been documented in other high-density island cities, where land scarcity has exacerbated transport problems and environmental inequalities [50].

In addition, in the case selection process, both geographic distribution and city size were considered, encompassing multicultural regions in Europe, the Americas, and the Asia-Pacific region, from mega-cities such as New York and Tokyo to medium-sized tourist cities such as Devonport, New Zealand, to examine tourism crowding conditions across different urban scales. Finally, the selected cases offered good data accessibility; most were well-established areas with abundant relevant statistics, planning documents, and academic outputs, facilitating theoretical support and field investigations. On the basis of these criteria, 28 representative urban areas were selected, as shown in Fig 1.

**Fig 1:**
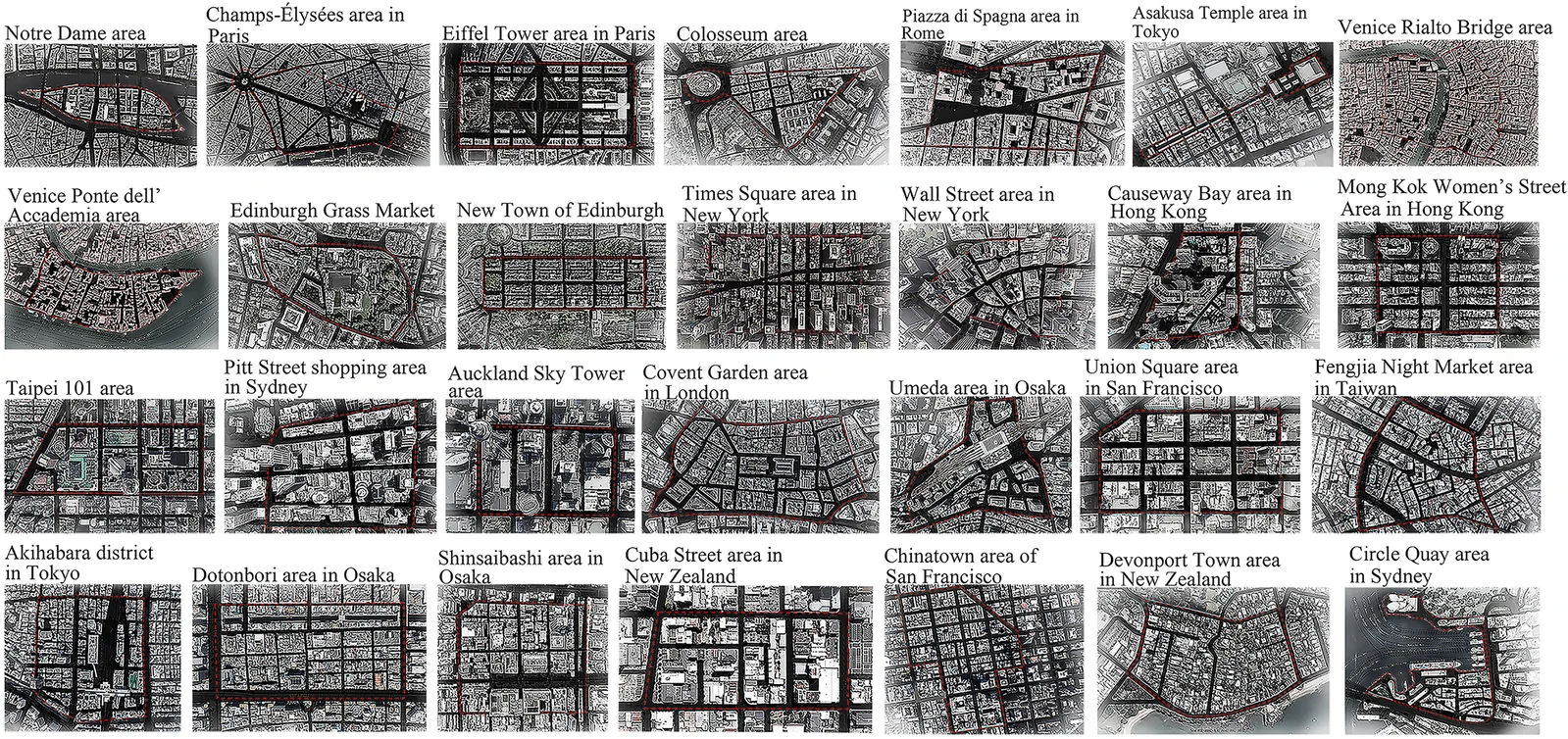
Overview of the study area (Base map source:Map data © OpenStreetMap contributors, available from https://www.openstreetmap.org, licensed under CC BY 4.0. Software used: Adobe Photoshop 2021, Adobe Illustrator 2022).

## 4 Results

### 4.1 Results from basic urban data

The data collected from each case are summarized in Tables 2 and 3. Analysis of physical crowding-related factors reveals that most tourist areas feature a well-developed road network and an open, multipath neighborhood structure, providing tourists with a variety of route choices. Only three exceptions are identified: the Eiffel Tower area in Paris, the Piazza di Spagna area in Rome, and the Asakusa Temple area in Tokyo. These three areas are characterized by the dominance of a single axial path. Specifically, the Eiffel Tower area in Paris and the Asakusa Temple area in Tokyo are both traversed by a longitudinal axis that guides visitors to core attractions, such as the Eiffel Tower and the main hall of Asakusa Temple. Although a few secondary roads connect the plaza in the Piazza di Spagna area, this area relies primarily on a major road serving both transportation and sightseeing, which limits route options for visitors. With respect to road width, the Champs-Élysées in Paris is the widest. The status of this region as a globally renowned boulevard necessitates traffic, sidewalk, and landscaped strip accommodations, which contribute to its large overall street scale. In contrast, the Asakusa Temple area in Tokyo features the narrowest roads; its core passageway, Nakamise Shopping Street, is a traditional area retaining the compact scale of the Edo period.

**Table 2:** Data display of Physical crowding-related factors.

Area name	Physical crowding-related factors							
	Selectivity of tourist paths	Road width	Road network density	Building coverage ratio	Building Spacing	Building count within the area	Vehicle count within the area	Site area (km^2^)
Champs- Élysées area in Paris	1	20.93	25.0	44.70%	10.83	63	2033	1.38
Notre Dame Area	1	8.711	19.7	42.03%	16.35	32	628	0.076
Eiffel Tower area in Paris	0	12.67	15.8	14.35%	24.48	32	392	0.49
Colosseum area	1	12.29	25.7	36.19%	12.83	53	1457	0.249
Spanish Steps area in Rome	0	8.402	32.0	58.40%	6.86	12	292	0.225
Asakusa Temple area in Tokyo	0	5.12	25.5	45.98%	3.635	18	0	0.012
Venice Rialto Bridge area	1	5.32	23.8	32.82%	3.47	78	0	0.189
Venice Ponte dell’Accademia area	1	5.83	25.1	34.27%	5.68	75	0	0.194
Edinburgh Grass Market	1	8.36	28.2	38.52%	9.49	31	281	0.144
New Town of Edinburgh	1	15.78	26.8	57.38%	7.98	41	671	0.383
Times Square area in New York	1	10.78	20.0	63.17%	16.55	29	1208	0.503
Wall Street area in New York	1	8.1	28.4	63.06%	11.49	23	387	0.167
Causeway Bay area in Hong Kong	1	8.51	31.3	59.71%	8.24	11	116	0.059
Mong Kok Women’s Street area in Hong Kong	1	5.97	30.2	53.88%	7.64	43	298	0.082
Taipei 101 area	1	16.15	18.9	25.64%	24.09	15	195	0.279
Pitt Street shopping area in Sydney	1	12.45	19.0	58.46%	13.96	8	209	0.178
Auckland Sky Tower Area	1	14.22	20.7	52.34%	10.79	8	121	0.0853
Covent Garden area in London	1	8.33	27.4	54.09%	6.31	44	388	0.267
Umeda area in Osaka	1	14.71	17.1	31.74%	19.8	33	607	0.529
Union Square area in San Francisco	1	11.67	22.4	57.03%	16.17	14	334	0.186
Chinatown area of San Francisco	1	7.46	21.0	56.48%	8.41	49	605	0.33
Akihabara District in Tokyo	1	13.81	30.2	51.75%	6.62	94	171	0.22
Dotonbori area in Osaka	1	6.32	32.2	74.22%	5.52	146	173	0.155
Shinsaibashi area in Osaka	1	6.48	28.5	54.92%	5.71	93	95	0.118
Fengjia Night Market area in Taiwan	1	9.27	31.2	70.23%	5.58	98	199	0.123
Cuba Street area in New Zealand	1	11.37	31.9	48.43%	5.31	28	319	0.0738
Devonport Town area in New Zealand	1	7.63	22.9	20.28%	5.87	126	195	0.161
Circle Quay area in Sydney	1	10.49	15.0	37.98%	8.37	30	149	0.22

**Table 3:** Data display of Perceived crowding-related factors.

Area name	Perceived crowding-related factors					
	Building configuration factor	Building height	Depth-to-height ratio (D/H)	Façade color	Green space ratio	Building height-to-width ratio
Champs- Élysées area in Paris	5.25	25.39	2.27	2.17	11.28%	1.02
Notre Dame Area	6.52	26.47	1.389	2.41	14.95%	0.485
Eiffel Tower area in Paris	3.87	22.19	4.67	2.83	29.06%	2.6
Colosseum area	5.606	21.75	0.674	2.17	13.02%	0.258
Spanish Steps area in Rome	3.01	22.29	1.705	3.25	2.44%	0.517
Asakusa Temple area in Tokyo	3.97	11.085	1.796	2.67	1.23%	1.007
Venice Rialto Bridge area	3.08	19.77	0.514	3.92	0.73%	0.301
Venice Ponte dell’Accademia area	2.87	18.63	0.911	3.75	17.39%	0.387
Edinburgh Grass Market	6.62	17.96	1.45	2.5	33.76%	0.254
New Town of Edinburgh	1.649	18.64	1.748	2.58	7.59%	0.112
Times Square area in New York	5.52	94.6	0.36	4.5	1.1%	0.638
Wall Street area in New York	7.738	92.36	0.43	2.16	0.96%	2.972
Causeway Bay area in Hong Kong	14.3	103.35	0.119	4.33	1.33%	1.495
Mong Kok Women’s Street area in Hong Kong	6.47	45.23	0.239	4.58	0	1.77
Taipei 101 area	1.77	119.09	1.35	4.08	7.9%	4.14
Pitt Street shopping area in Sydney	11.15	115.15	0.715	1.92	2.37%	0.695
Auckland Sky Tower Area	11.29	100.86	0.676	2.92	1.37%	0.632
Covent Garden area in London	3.61	22.26	0.986	3.75	2.74%	0.214
Umeda area in Osaka	2.252	94.61	0.2	2.83	5.97%	0.98
Union Square area in San Francisco	5.55	49.83	0.324	2.58	2.20%	0.409
Chinatown area of San Francisco	2.206	43.67	1.78	3.92	4.16%	0.109
Akihabara District in Tokyo	2.5	74.39	1.049	4.67	2.16%	0.762
Dotonbori area in Osaka	11.79	25.17	0.314	4.5	1.37%	0.929
Shinsaibashi area in Osaka	10.41	36.74	0.955	2.75	4.97%	0.458
Fengjia Night Market area in Taiwan	1.011	17.48	0.973	4.58	4.80%	0.357
Cuba Street area in New Zealand	3.18	13.53	0.877	3.92	3.91%	0.304
Devonport Town area in New Zealand	0.92	6.4	1.28	1.67	68.05%	0.195
Circle Quay area in Sydney	2.17	19.23	1.215	1.91	16.94%	0.294

Renowned as a bustling commercial area, Dotonbori boasts the highest road network density. This dense road network enhances commercial value and attracts tourists. In contrast, Sydney’s circular quay area has the lowest road network density. Constrained by topography, this area lacks the dense network typical of commercial districts, resulting in relatively few roads. In terms of building coverage, the Fengjia Night Market area in Taiwan has the highest density of commercial buildings. Conversely, the area surrounding the Eiffel Tower in Paris has the lowest density and is characterized by expansive green spaces and squares, scattered buildings, and the greatest building spacing. The Asakusa Temple area in Tokyo features the smallest building spacing, particularly along Nakamise Shopping Street, where stores are closely packed, preserving the traditional street scale. The Dotonbori area in Osaka boasts the highest building count, consisting mainly of numerous small stores, whereas Sydney’s Pitt Street area has the fewest buildings, as it is dominated by large shopping centers and few standalone structures. In terms of vehicle count, the Champs-Élysées in Paris has the greatest volume because of its expansive site area and wide roads, which accommodate large numbers of vehicles for both passage and parking. Conversely, Asakusa Temple in Tokyo and the Rialto and Ponte dell’Accademia area in Venice have no vehicular traffic. Asakusa Temple enforces a no-vehicle policy, whereas Venice relies on its waterways, with transportation occurring mainly on foot or by boat.

On the basis of the analysis of perceived crowding-related factors, Causeway Bay in Hong Kong has the highest building configuration factor. As one of Hong Kong’s most densely populated commercial districts, Causeway Bay features high-rise shopping malls and residential and office buildings, and it presents complex, varied architecture. The lowest building configuration factor is observed in the area of Devonport, New Zealand, which is characterized by low-rise single-family homes and small stores, resulting in a simpler, less varied overall form. In terms of building height, Times Square in New York stands out as the tallest area, as it is home to numerous super-tall buildings (e.g., One Times Square and Condé Nast Tower), which contribute to a markedly high average height. As a global center of commerce and entertainment, this area faces severe land scarcity, which drives vertical development. Conversely, the lowest building heights are observed in the area of Devonport, where a slow-living atmosphere is prioritized, traditional villages are preserved, and building heights are strictly regulated because of historic preservation policies.

The Eiffel Tower precinct in Paris has the highest depth-to-height ratio (D/H). This zone features a central plaza as its core, which is characterized by expansive open space and low-rise buildings, creating a pronounced sense of spatial openness. The area with the lowest D/H is Causeway Bay in Hong Kong, which is characterized by narrow streets such as Lockhart Road and flanked by high-rise buildings, resulting in a significant sense of oppression. In terms of façade color, the Akihabara district in Tokyo scores the highest. As a hub of otaku culture, the area is adorned with numerous anime posters, game characters, light-emitting diode screens, and other vibrant displays, creating extremely high color saturation and a striking visual impact. Conversely, the lowest-scoring area is the Devonport town area, where buildings predominantly feature muted colors such as beige and light gray, creating a uniform color palette. The abundant greenery in Devonport softens the visual impact of these plain colors. Furthermore, Devonport boasts the highest green space ratio, as it is surrounded by mountains and the sea and features numerous natural green areas and parks. In contrast, the Times Square area in New York and Mong Kok Women’s Street in Hong Kong exhibit the lowest green space ratios. Both locations are high-density commercial districts, where the landscape is dominated by buildings and hard surfaces, leading to a significant lack of greenery.

### 4.2 Clustering results of crowding types

To verify the independence of the indicators, a multicollinearity test was conducted before correlation analysis was performed. The results reveal that the variance inflation factor (VIF) of each variable is less than 5, indicating that no severe multicollinearity problem exists in the indicator system, thereby ensuring the reliability of the subsequent cluster and correlation analyses.

The elbow method plot shows that the WCSS curve decreases steeply from k = 2 to k = 3, and the rate of decrease declines markedly from k = 3 to k = 4. For k ≥ 4, the WCSS values change only marginally, with no meaningful improvement (Fig 2). In the Calinski–Harabasz index (CHI) plot, the CHI value reaches a local maximum at k = 4, indicating a favorable trade-off between intercluster dispersion and intracluster compactness. An excessive number of clusters would result in some clusters with too few samples and an overly fragmented typology, reducing the interpretability and practical utility of the classification. Accordingly, k = 4 is chosen as the most reasonable number of clusters (Fig 1). Thus, k = 4 achieves an optimal balance between cluster separation and intracluster cohesion, providing converging evidence with the elbow method and the CHI.

**Fig 2:**
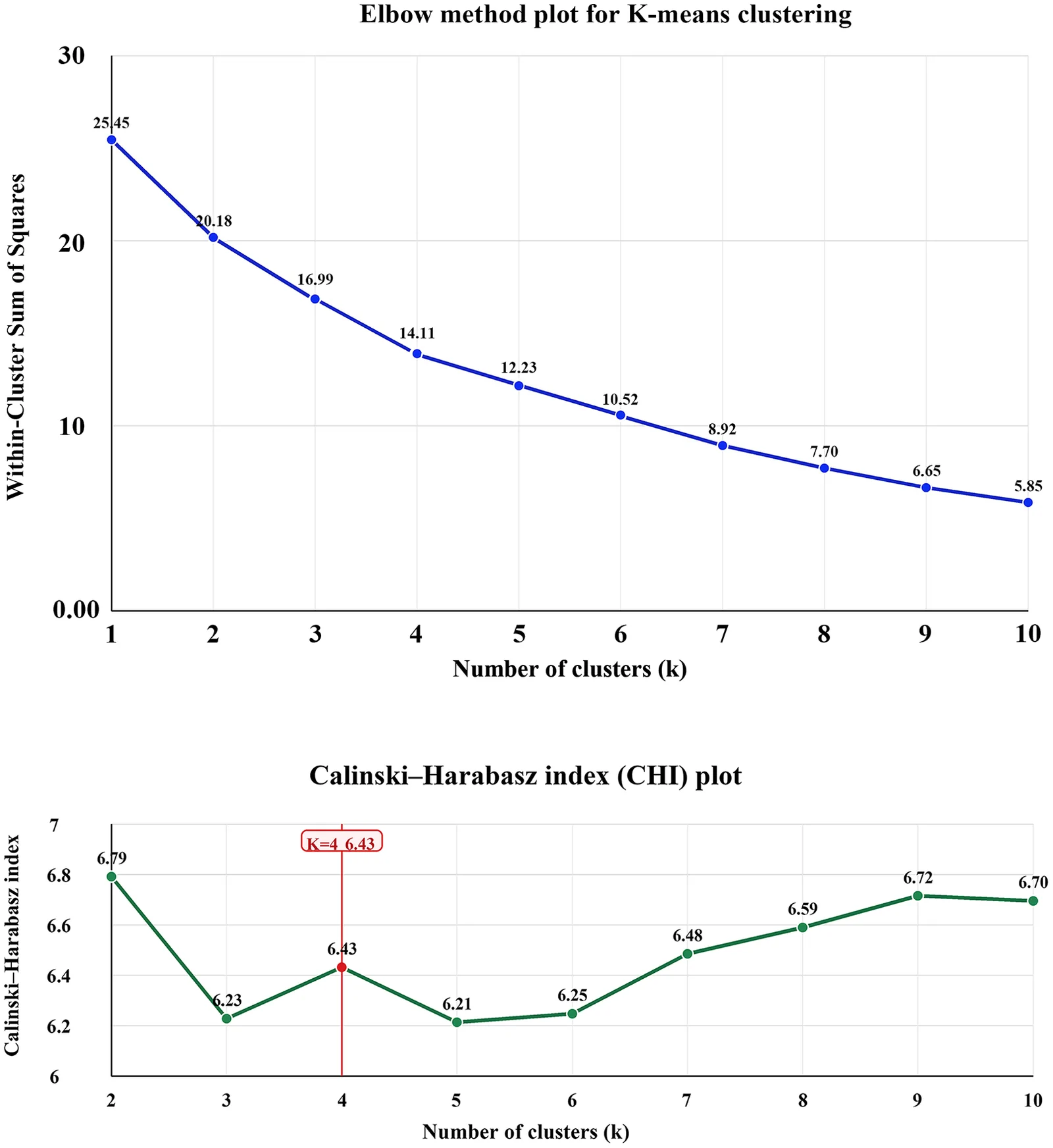
Cluster number analysis diagram. (Figure source: drawn by the author. Software used: SPSSAU, python).

The collected data were imported into SPSS for hierarchical clustering, and the cluster assignment was determined from the dendrogram with the number of clusters set to four. The details are presented in Fig 3.

**Fig 3:**
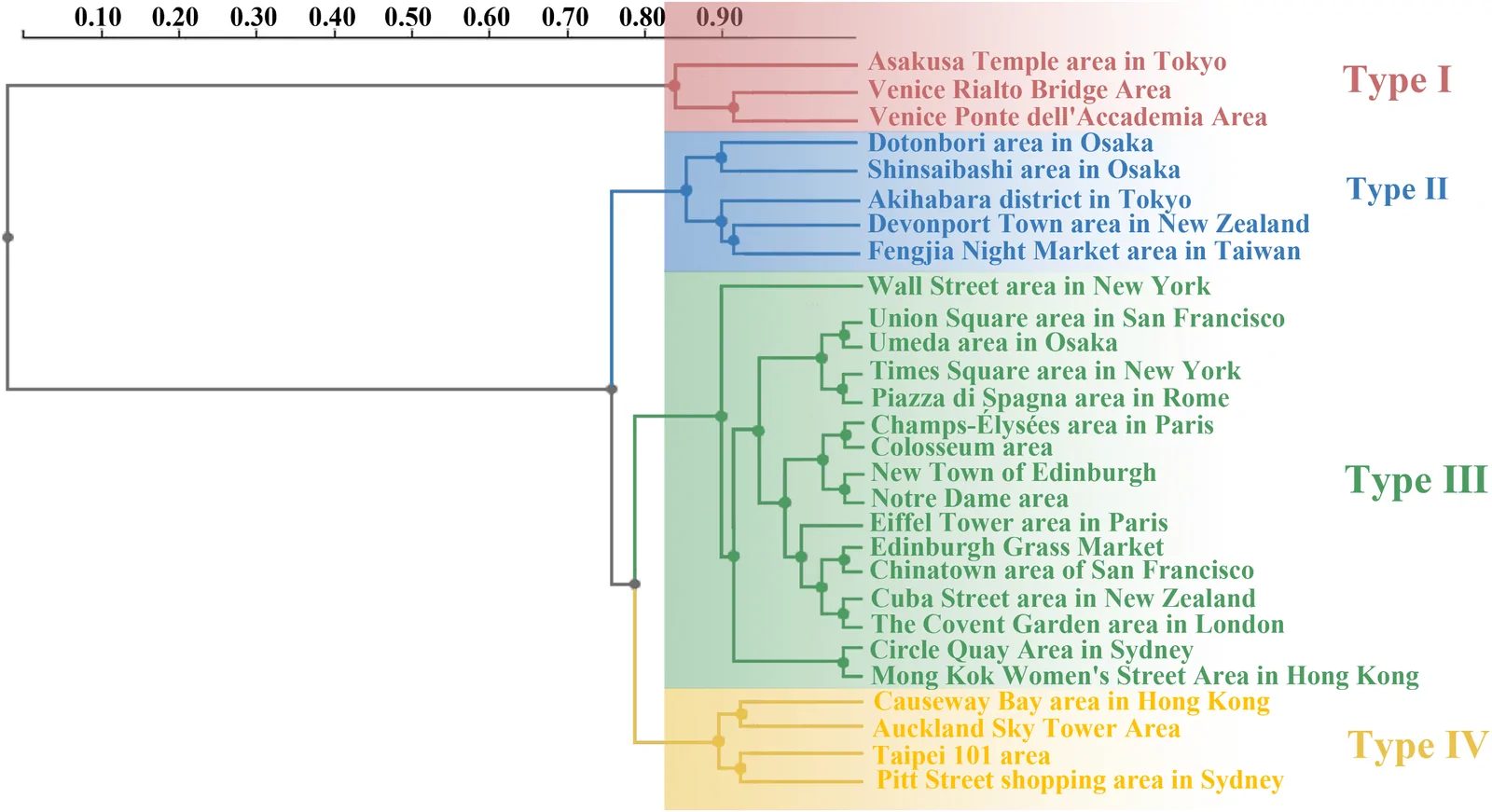
Clustering dendrogram. (Figure source: drawn by the author. Software used: SPSSAU, Adobe Photoshop 2021, Adobe Illustrator 2022).

Type Ⅰ: Asakusa Temple area in Tokyo, Venice Rialto Bridge area, and Venice Ponte dell’Accademia area.

Type II: Devonport Town area in New Zealand, Fengjia Night Market area in Taiwan, Akihabara district in Tokyo, Dotonbori area in Osaka, and Shinsaibashi area in Osaka.

Type III: Champs-Élysées area in Paris, Eiffel Tower area in Paris, Union Square area in San Francisco, Chinatown area of San Francisco, Umeda area in Osaka, Cuba Street area in New Zealand, Colosseum area, Circle Quay area in Sydney, Mong Kok Women’s Street area in Hong Kong, Edinburgh Grass Market, New Town of Edinburgh, Covent Garden area in London, Times Square area in New York, Piazza di Spagna area in Rome, Notre Dame area, and Wall Street area in New York.

Type Ⅳ: Causeway Bay area in Hong Kong, Taipei 101, Auckland Sky Tower Area, and Pitt Street shopping area in Sydney.

## 5 Discussion

### 5.1 Classification of crowding types

In this study, a hierarchical cluster analysis was conducted using SPSS to classify 28 high-density tourist urban areas into four distinct crowding perception types that differ significantly from one another. This classification reveals the mechanisms through which different spatial features influence crowding perceptions. Notable examples of the first type include the Asakusa Temple area in Tokyo, the Rialto Bridge area in Venice, and Venice Ponte dell’Accademia area. The results of the analysis indicate that these areas share common spatial characteristics, such as very narrow building spacings and road widths (Fig 4). This type of spatial configuration can be defined as a closely spaced narrow-alley type, which induces crowding through a constrained spatial scale. Dense building rows heighten the feeling of spatial oppression [28].

**Fig 4:**
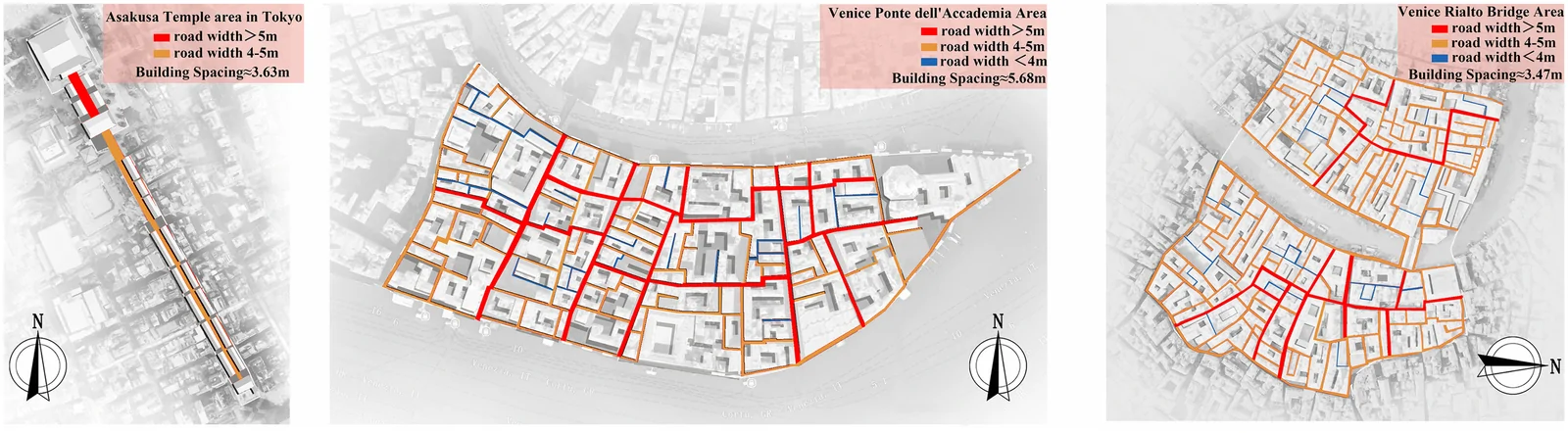
Closely spaced narrow alley-type diagram. (Base map source:Map data © OpenStreetMap contributors，available from https://www.openstreetmap.org, licensed under CC BY 4.0. Software used: Adobe Photoshop 2021, Adobe Illustrator 2022, QGIS).

Moreover, road width directly affects accessibility. Wider roads can theoretically accommodate more pedestrian and vehicular traffic, thereby alleviating crowding [41], whereas narrower roads restrict flow, create oppression, and reduce access efficiency. This characteristic, in turn, exacerbates tourist stranding and perceived crowding. Additionally, such areas are distinctly nonmotorized, with no vehicular traffic being recorded. Their narrow street scale is unsuitable for motor vehicles, making walking the primary travel mode.

The second type comprises the Devonport Town area in New Zealand, the Fengjia Night Market area in Taiwan, Akihabara in Tokyo, Dotonbori in Osaka, and the Shinsaibashi area in Osaka. According to the quantitative data, this group has a significantly higher building count than the other types do. This spatial configuration can be defined as the high-building-quantity type, characterized by a sense of crowding arising from excessive construction (Fig 5). When the ground level is occupied by numerous buildings, it significantly affects users’ emotional states, thereby exacerbating perceived crowding [36]. In contrast to the Fengjia Night Market area, Akihabara area in Tokyo, Dotonbori area in Osaka, and Shinsaibashi area in Osaka, all of which are renowned commercial districts with high building densities and substantial building coverages, Devonport Town, although similarly categorized, exhibits markedly distinct spatial characteristics. This area primarily emphasizes ecological recreation and cultural experiences, with buildings predominantly being low-rise or multistory and average building heights that are the lowest among the studied areas. The spatial structure is characterized by horizontal expansion, with small monolithic volumes and a dispersed layout, resulting in a high overall number of buildings.

**Fig 5:**
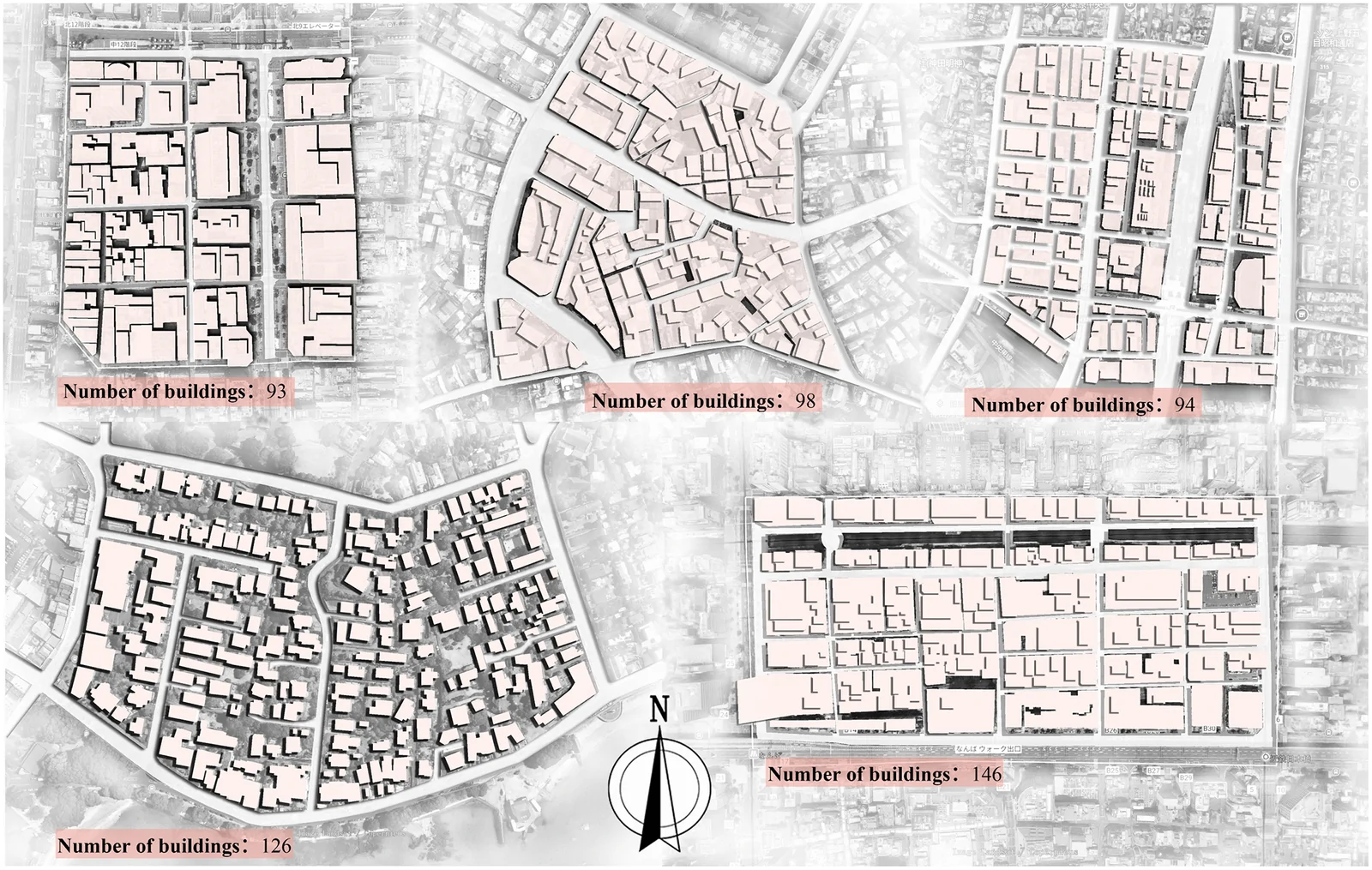
High building quantity type diagram. (Base map source:Map data © OpenStreetMap contributors, available from https://www.openstreetmap.org, licensed under CC BY 4.0. Software used: Adobe Photoshop 2021, Adobe Illustrator 2022, QGIS).

The fourth type encompasses the Causeway Bay area in Hong Kong, Taipei 101, the Auckland Sky Tower area, and the Pitt Street shopping area in Sydney. All these locations are bustling commercial districts in the urban core. These areas exhibit two prominent spatial characteristics: a high concentration of supertall buildings and predominantly hard-paved surfaces with minimal green space. Consequently, the areas can be classified as the high-rise low-greenery type. Research has shown that compared with the horizontal dimension, the vertical dimension of buildings has a significantly greater effect on psychological oppression [28]. Moreover, the absence of green spaces exacerbates this sense of oppression. Introducing vegetation not only helps alleviate anxiety, stress, and depression but also effectively reduces perceived spatial crowding [36,37]. Notably, in such areas, the building count is relatively low, whereas the building spacing is relatively high because of the increased building heights.

The remaining cases are classified as the third type, which, on the basis of the data, presents a markedly greater number of motor vehicles and is defined as the high‑vehicle‑interference type. The presence of motor vehicles is a major negative factor in evaluating urban environments, and high vehicle interference significantly exacerbates perceived crowding and negative emotions [37]. Specifically, an excessive number of vehicles may lead to noise pollution, traffic conflicts, and an increased risk of accidents. Notably, some areas within Type IV cases face challenges such as narrow road widths (e.g., Women’s Street in Mongkok, Hong Kong) and low road network density (e.g., Circular Quay, Sydney). The combination of restricted road space, low road network density, and a high volume of motor vehicles tends to aggravate traffic problems and further intensify perceived crowding.

### 5.2 Correlation analysis of the influential elements of crowding perception

In this study, the interaction mechanisms among factors are revealed through Pearson correlation analysis. The results indicate both significant positive and negative correlations among several factors. The significantly correlated factor pairs and their potential causes are explained below (Table 4 and Fig 6). Notably, a significant positive correlation exists between road network density and building coverage. An increase in building coverage is often accompanied by an increase in population and functional density, thereby heightening the demand for transportation accessibility. The operational performance of urban road networks largely depends on their structural configuration; a dense road network can reduce travel distances and enhance network connectivity, thereby supporting transportation efficiency in high-density development areas [51]. Furthermore, high building coverage indicates that the land is densely occupied by structures. To accommodate infrastructure needs such as transportation and fire protection, planning must subdivide large land areas into relatively small parcels through a dense road network [52]. Within a specific land area, building spacing is significantly negatively related to both the degree of building coverage and the number buildings. An increase in building spacing directly reduces the building footprint per unit area while decreasing the building capacity of the site. Conversely, reducing building spacing can theoretically increase building density, thereby increasing the number of buildings and overall coverage [40]. As a crucial indicator of spatial enclosure, the D/H ratio directly influences users’ spatial perception; a larger D/H value corresponds to a more spacious environment [31]. However, increased spatial openness inevitably reduces building coverage. Given a fixed land area, the green space ratio and building area are in a competitive relationship. An increase in the green space ratio inevitably leads to a decrease in building coverage, whereas an increase in building coverage correspondingly decreases the green space ratio.

**Table 4:** Interindicator Pearson correlation matrix for crowding measures.

		Building coverage ratio	Building count within the area	Vehicle count within the area	Building configuration factor	Building height	Façade color	Building height-to-width ratio
Selectivity of tourist paths	Correlation coefficient	0.197	0.258	0.140	0.156	0.262	0.106	−0.188
	P value	0.316	0.185	0.476	0.428	0.178	0.590	0.338
Road width	Correlation coefficient	−0.169	−0.261	0.577**	−0.129	0.339	−0.225	0.213
	P value	0.390	0.180	0.001	0.514	0.077	0.250	0.278
Road network density	Correlation coefficient	0.556**	0.331	−0.132	0.197	−0.259	0.429*	−0.149
	P value	0.002	0.085	0.503	0.314	0.184	0.023	0.450
Building spacing	Correlation coefficient	−0.386*	−0.452*	0.298	−0.011	0.500**	−0.187	0.571**
	P value	0.042	0.016	0.124	0.956	0.007	0.340	0.001
Site area (km^2^)	Correlation coefficient	−0.169	0.029	0.789**	−0.202	0.001	−0.175	0.088
	P value	0.391	0.884	0.000	0.304	0.995	0.374	0.658
Depth-to-Height ratio (D/H)	Correlation coefficient	−0.472*	−0.069	0.154	−0.338	−0.375*	−0.261	0.186
	P value	0.011	0.727	0.433	0.078	0.049	0.181	0.343
Green space ratio	Correlation coefficient	−0.646**	0.310	0.003	−0.294	−0.383*	−0.472*	−0.102
	P value	0.000	0.108	0.989	0.129	0.044	0.011	0.606

Note: * *p* < 0.05 ** *p* < 0.01.

**Fig 6:**
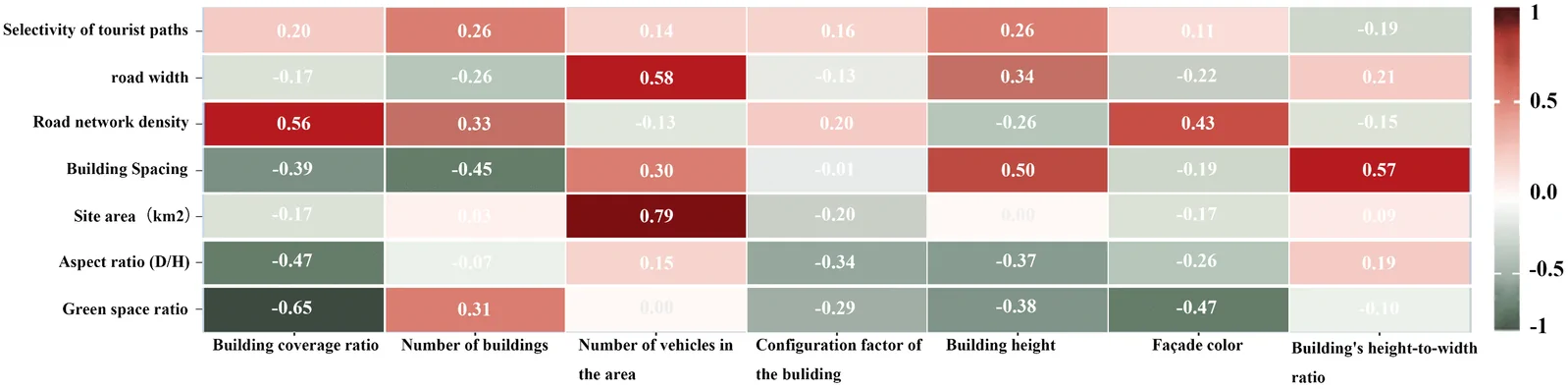
Correlation analysis chart. (Figure source: drawn by the author. Software used: SPSSAU, Adobe Photoshop 2021).

Significant positive correlations exist among the building spacing, height, and height-to-width ratio. In accordance with the current Code for Fire Protection Design of Buildings (GB50016-2014), a building’s fire separation distance must take its height into account. Generally, the required fire separation distance is positively correlated with building height. For instance, buildings taller than 50 meters require a minimum distance of 13 meters, whereas low‑rise buildings require only 9 meters. Additionally, to ensure adequate natural lighting, building spacing must be proportionally increased with building height to prevent mutual shading. The height-to-width ratio of a building is a crucial indicator of its form characteristics. A higher height-to-width ratio typically results in a slenderer and taller architectural form. Consequently, the building spacing should be positively correlated with the height-to-width ratio.

Façade color is positively correlated with road network density, as a dense road network broadens the visual display space of stores by increasing the length of street interfaces and commercial exposure surfaces. This phenomenon is particularly noticeable in high-intensity development areas, such as commercial cores, where stores construct a high-contrast, high-saturation street color landscape through visual symbol systems such as façade advertisements, artistic graffiti, and dynamic neon [53]. Therefore, the higher the road network density is, the richer the color in the spatial scene. However, façade color is negatively correlated with green space. In terms of environmental psychology, green space is a vital component of urban ecosystems and plays a key role in stress reduction and anxiety relief [54]. Low-saturation natural colors (e.g., lab values for green hues typically range from 50 ± 15, −20 ± 10, and 30 ± 15) exert a buffering–neutralizing effect on artificial colors. Consequently, the greater the amount of green vegetation in a scene is, the less oppressive the perceived color of that scene.

The number of vehicles within the area is significantly positively correlated with both the road width and site area. In terms of traffic capacity, an increase in road width directly increases the number of vehicles that can pass through per unit of time, thereby accommodating a greater volume of traffic. With respect to parking, a wider road cross-section allows for more on-street parking. For instance, a 12‑meter‑wide road can support double‑sided parking, whereas a 9‑meter‑wide road reduces the parking capacity by approximately 50%. Similarly, a larger site area provides more space for improved parking facility layouts, and increases in site area are typically accompanied by the expansion of road networks and parking facilities.

### 5.3 Limitations

In terms of sample representativeness, in future research, scholars should significantly expand the sample size and include more representative high-density urban areas to validate the generalizability and scientific validity of these findings. With respect to the construction of the indicator system, existing influencing factors are focused mainly on physical spatial elements. In future research, scholars should incorporate multidimensional indicators, such as socioeconomic and cultural background factors, to comprehensively reflect the complex causes of perceived crowding. With respect to data timeliness, static data cannot accurately capture dynamic traffic flows and temporary spatial usage behaviors. This study relied primarily on Google Maps data and questionnaire surveys; in subsequent research, multisource spatiotemporal big data, such as mobile phone signaling data and social media data, should be integrated to construct a spatiotemporal fusion analytical model. In addition, the measurement of the color oppressiveness of building façades in this study still relies on traditional subjective questionnaires. Researchers can utilize computer vision and image semantic segmentation techniques to extract objective quantitative indicators, such as color saturation and contrast, from street‑view images and cross‑validate them with tourists’ subjective ratings, thereby enhancing the robustness and objectivity of the perceptual dimension data.

Furthermore, building on the crowding type classification system established in this study, scholars should formulate differentiated spatial optimization strategies tailored to the characteristics of each crowding type. Specifically, by integrating space syntax theory, environmental psychology, and related methods, targeted design guidelines and implementation pathways can be proposed for different crowding types, such as the closely spaced narrow alley type and the high building quantity type, to improve the quality of the urban environment. Considering the impact of tourism gentrification on urban space use, subsequent studies should adopt a multiuser perspective, distinguishing perceptual differences among groups such as permanent residents and tourists. By constructing a multidimensional evaluation framework, the differences in adaptability to urban crowding among various populations can be quantitatively analyzed, thereby providing a basis for more inclusive urban planning.

## 6 Conclusion

In this study, the crowding types and influences of 28 high-density tourist city areas worldwide were analyzed from the dual dimensions of physical and perceived density. The main conclusions were as follows. First, the cluster analysis based on spatial morphology characteristics revealed that the crowding phenomenon in high-density urban areas could be classified into four typical types: (1) closely spaced narrow alley type, characterized by narrow building spacing and road width, which significantly increased crowding perception through enhanced spatial enclosure and reduced access efficiency; (2) high building quantity type, where excessive construction within the area led to a crowded feeling; (3) high vehicle interference type, where greater vehicle interference triggered negative emotions and thereby increased crowding perception; and (4) high-rise low-greenery type, where super-high-rise buildings and a very low green space ratio intensified the sense of spatial oppression and increased crowding perception. Second, correlation analysis revealed the interaction mechanisms among factors. Building coverage was positively correlated with road network density. The building spacing was positively correlated with both the building height and height-to-width ratio. The color of the façade was positively correlated with the road network density. The vehicle count within the area was positively correlated with both the road width and site area. Conversely, building coverage was negatively correlated with the building spacing, depth-to-height ratio (D/H), and green space ratio. These findings have rarely been reported in existing studies. Through empirical analysis, this research led to improvements in the evaluation indicators in terms of both physical and perceived dimensions and the identification of four typical types of congested spaces. Furthermore, this research provides a solid theoretical and empirical basis for developing additional targeted urban planning strategies in the future.

## Supporting information

S1 File Basic data. The original data used for the clustering and correlation analyses.
(XLSX)
